# Management of a Spontaneous Carotid Artery Blowout

**Published:** 2019-02-21

**Authors:** Shabaaz S. Sandhu, Jake Laun, Joshua B. Elston, Nicholas J. Panetta

**Affiliations:** Department of Plastic Surgery, University of South Florida Morsani College of Medicine, Tampa

**Keywords:** carotid artery rupture, pectoralis major, neck dissection, reconstruction, radiation

## DESCRIPTION

A 71-year-old man awoke in the night to spontaneous and profuse bleeding from his neck 17 years after neck dissection and radiation therapy to treat squamous cell carcinoma. Vascular stents were emergently placed in the ruptured and exposed carotid artery. Plastic Surgery was consulted for assistance with wound coverage.

## QUESTIONS

1. What is carotid blowout syndrome (CBS) and how is it diagnosed?
2. What are the risk factors that predispose to development carotid artery blowout?
3. How is carotid blowout treated?
4. What is the prognosis of CBS?

## DISCUSSION

CBS represents the rupture of the carotid artery or one of its main branches and is a serious complication of head and neck cancer or sequela of its therapy.^1^ Clinical presentations range from asymptomatic exposure of the carotid artery wall to acute and possibly life-threatening hemorrhage.^2^ CBS can be classified into 1 of 3 categories: threatened, impending, and acute carotid blowout. Threatened carotid blowout is characterized by physical and radiological evidence suggestive of inevitable hemorrhage. Impending carotid blowout is an episode of hemorrhage that resolves spontaneously, with packing, or pressure. Acute carotid blowout represents hemorrhage that cannot be stopped by packing or pressure.^3^ Angiography is the gold standard for the diagnosis of CBS, which most often occurs at the internal carotid artery (43.6%), external carotid artery (23.4%), or common carotid artery (11.7%).^3^^,^^4^ CBS has a prevalence of 3.9% in patients with head and neck cancer who underwent definitive treatment, with mortality and major neurological morbidity rates of 40% and 60%, respectively.^3^^,^^4^

CBS develops when a damaged arterial wall cannot sustain its integrity against the patient's blood pressure.^2^ The most common etiology of CBS is squamous cell carcinoma or its treatment. Irradiation has been associated with a 7-fold increase in the risk of CBS in patients with head and neck cancer.^3^ Total radiation dose in the neck is the single most significant predisposing factor in this population—patients who received a total radiation dose of 70 Gy or more to the neck carried a near 14-fold increase of developing carotid blowout.^2^ It is thought that direct radiation injury to the adventitial layer renders the artery more vulnerable to acute rupture, whereas delayed rupture may be due to soft tissue fibrosis and progressive hypoxia.^1^^,^^2^ CBS develops an average of 2.7 years after the initial diagnosis of cancer, with a mean duration of 23 months from initial treatment to the first carotid blowout.^2^ Neck surgery (particularly in the context of pharyngocutaneous fistula formation), infection, and poor nutrition can also predispose patients to carotid exposure and ultimate rupture.^1^^,^^2^

Historically, open surgical intervention with arterial repair or ligation was the favored treatment modality.^1^ Because of the higher mortality and morbidity rate associated with surgical management, endovascular therapy (including embolization and stent grafting) has become the standard of care.^4^ In the case of significant carotid exposure without a history of bleeding, the focus of treatment is coverage of the artery with vascularized tissue. In patients with threatened and impending carotid blowout, angiography is required and should be both diagnostic and therapeutic.^3^ In the case of acute carotid blowout, hemorrhage must first be controlled before proceeding with endovascular management.^1^ Carotid ligation of the involved vessel should be seen as a last resort, as it has been associated with a higher risk of neurological sequelae, wound-healing problems, and rehemorrhage.^3^

CBS is a spectrum of presentations of damage to the carotid artery along its length in the setting of prior therapy for head and neck cancer. Radiation therapy as well as the total dose of radiation given can increase the risk of occurrence. Angiography is useful in diagnostic and therapeutic management, with flap reconstruction being necessary for severe cases or those requiring more durable soft tissue coverage. As with all massive hemorrhagic events, rapid initial resuscitation and prompt management of the source of bleeding will improve patient survival. Recurrence rate is unknown; however, median survival after CBS is 12 months.^4^ Patients and families should be counseled about the high neurological morbidity and possible mortality rate associated with CBS.

## Figures and Tables

**Figure 1:**
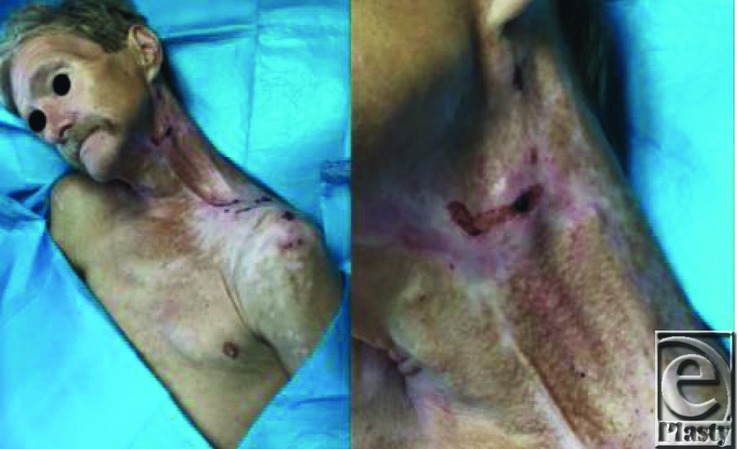
On presentation of spontaneous carotid artery rupture after a remote history of neck dissection and radiation therapy. Note the dark spot at the rupture site.

**Figure 2:**
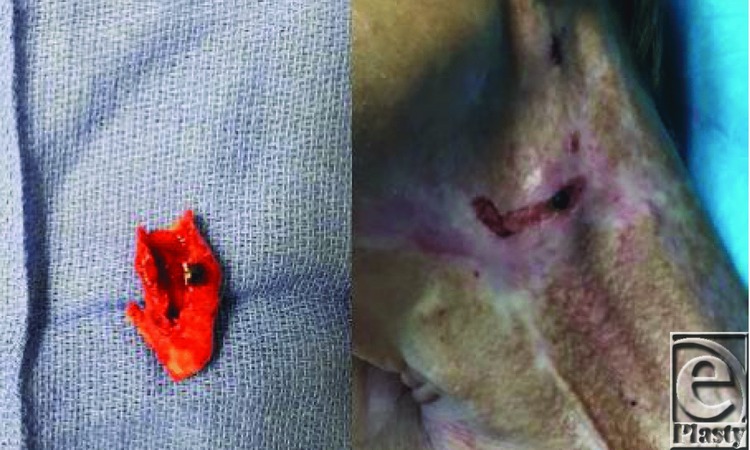
Portion of carotid artery removed and ultimately bypassed with a vein graft. Note the corresponding black spot on clinical examination.

**Figure 3:**
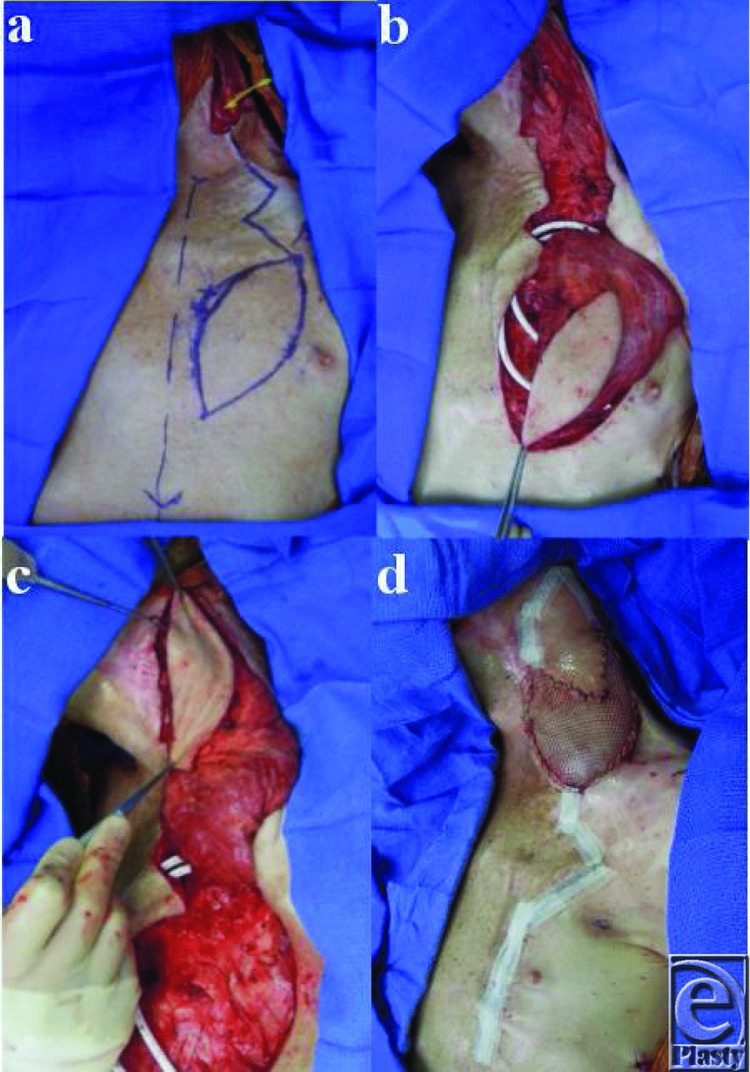
(*a*) After removal of ruptured carotid, interposition venous bypass grafting is performed and a flap is designed for coverage incorporating a skin paddle. (*b*) Myocutaneous pectoralis major dissected free. (*c*) Flap transposed into position. (*d*) Flap inset with meshed split-thickness skin graft for coverage of remaining muscle. Myocutaneous component of the flap covers the venous interposition graft.

**Figure 4:**
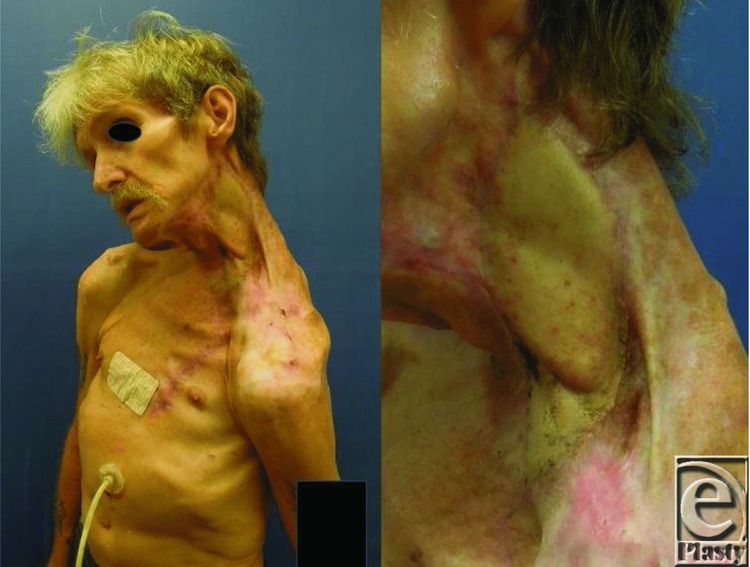
Follow-up at 4 months shows the well-healed flap.
